# Genome-Wide Identification of the *Eucalyptus urophylla* *GATA* Gene Family and Its Diverse Roles in Chlorophyll Biosynthesis

**DOI:** 10.3390/ijms23095251

**Published:** 2022-05-08

**Authors:** Kang Du, Yufei Xia, Dingju Zhan, Tingting Xu, Te Lu, Jun Yang, Xiangyang Kang

**Affiliations:** 1 National Engineering Research Center of Tree Breeding and Ecological Restoration, College of Biological Sciences and Technology, Beijing Forestry University, Beijing 100083, China; dukang@bjfu.edu.cn (K.D.); xiayufei@bjfu.edu.cn (Y.X.); tingtingxu0411@163.com (T.X.); 2 Key Laboratory of Genetics and Breeding in Forest Trees and Ornamental Plants, MOE, Beijing Forestry University, Beijing 100083, China; 3 The Tree and Ornamental Plant Breeding and Biotechnology Laboratory, National Forestry and Grassland Administration, Beijing Forestry University, Beijing 100083, China; 4 Guangxi Bagui R&D Institute for Forest Tree and Flower Breeding, Nanning 530025, China; dingju.zhan@gmail.com; 5 Science and Technology Section, Chifeng Research Institute of Forestry Science, Chifeng 024000, China; lute812885861@bjfu.edu.cn

**Keywords:** GATA, *Eucalyptus*, evolution, transcription factors, photosynthesis

## Abstract

GATA transcription factors have been demonstrated to play key regulatory roles in plant growth, development, and hormonal response. However, the knowledge concerning the evolution of *GATA* genes in *Eucalyptus urophylla* and their trans-regulatory interaction is indistinct. Phylogenetic analysis and study of conserved motifs, exon structures, and expression patterns resolved the evolutionary relationships of these GATA proteins. Phylogenetic analysis showed that *EgrGATAs* are broadly distributed in four subfamilies. *Cis*-element analysis of promoters revealed that *EgrGATA* genes respond to light and are influenced by multiple hormones and abiotic stresses. Transcriptome analysis revealed distinct temporal and spatial expression patterns of *EgrGATA* genes in various tissues of *E. urophylla* S.T.Blake, which was confirmed by real-time quantitative PCR (RT-qPCR). Further research revealed that *EurGNC* and *EurCGA1* were localized in the nucleus, and *EurGNC* directly binds to the *cis*-element of the *EurGUN5* promoter, implying its potential roles in the regulation of chlorophyll synthesis. This comprehensive study provides new insights into the evolution of *GATA*s and could help to improve the photosynthetic assimilation and vegetative growth of *E. urophylla* at the genetic level.

## 1. Introduction

GATA transcription factors are a class of genes with type IV zinc finger conserved structural domains (C-X2-C-X17-20-C-X2-C) and are widely present in various plants and animals [1]. Type IV zinc finger structures in plants are usually present as C-X2-C-X18-C-X2-C or C-X2-C-X20-C-X2-C [2]. Based on their evolutionary relationship and gene structure, *GATA* family genes can be divided into four subgroups [3]. As a transcription factor, it mainly regulates the expression of downstream genes by binding to conserved sequences, thus affecting the biological phenotype. The normally recognized consensus sequence of the *GATA* transcription factor family is WGATAR (W stands for T or A, and R stands for G or A). Analysis of the GATA family information on the JASPAR plant database revealed that 11 *GATA* genes have been sequenced (Dap-seq or ChIP-seq) or experimented with to verify their consensus sequence (GATC). The first *GATA* gene was identified in tobacco [4]. To date, *GATA* members have been reported in numerous plant species, among which 30 *GATA* transcription factors have been identified in *Arabidopsis*, and 28, 64, 179, and 262 *GATA* genes have been identified in the genomes of rice (*Oryza sativa*), soybean (*Glycine max*), cotton (*Gossypium hirsutum*), and seven poplars (*Populus*), respectively [2,5,6,7].

GATA transcription factors are widely involved in the biological processes of plant growth, development, and stress resistance. In common wheat, the GATA family transcription factor *ZIM-A1* can recognize the consensus sequence (TCKAG) in the promoter regions of the flowering genes, FLOWERING LOCUS T (*FT*) and CONSTANS (*CO*), and inhibit their expression [8]. GATA transcription factors are essential components of endogenous hormone pathways that govern a wide range of plant physiological processes. In *Arabidopsis*, *GATA12*, which has been identified as a downstream response factor of the DELLA protein *RGL2*, is inhibited by gibberellin and participates in the regulation of seed dormancy [9]. The *GATA7* contributes to the regulation of plant shape and grain type in rice via regulation of brassinosteroids [10]. According to previous research, *GATA23*, which is driven by several auxin response factors (*ARF*s), is involved in the formation of lateral roots and the regeneration of roots [11]. In addition, *ARF2* also directly affects the expression of GATA NITRATE-INDUCIBLE CARBON-METABOLISM-INVOLVED (*GNC*) and CYTOKININ-RESPONSIVE GATA FACTOR 1 (*GNL/CGA1*), thus regulating greening, flowering, and leaf senescence. Cytokinin can promote the expression of *GNL/CGA*, and gene mutations can result in the blocking of cytokinin-regulated growth processes, such as leaf senescence, hypocotyl growth, and branch formation [12]. Hence, *GNL/CGA* plays a key role in plant greening as it forms the junction between auxin and cytokinin [13].

Eucalypts are the main suppliers of high-quality woody biomass for the fiber, energy, and paper industry, with the added benefit of absorbing significant amounts of atmospheric carbon [14]. Improving the photosynthesis efficiency of *Eucalyptus* is of great significance for enhancing biomass accumulation and solving global warming problems. Chlorophylls, in conjunction with their binding proteins, play a key role in photosynthesis by capturing light energy and transmitting it to photosystem reaction centers [15,16]. To date, GATA transcription factors from a lot of species have been reported to control chloroplast development and chlorophyll synthesis [17]. In *Arabidopsis*, *GNC* and *GNL/CGA1* accelerated chloroplast development and greening during photomorphogenesis by directly targeting transcription factor (TF) genes, such as PHYTOCHROME-INTERACTING FACTOR (*PIF*) and SPEECHLESS (*SPCH*) [13,18,19]. Overexpression of *OsCGA1* or *OsGATA12* in rice increased chloroplast biogenesis and chlorophyll content and inhibited the expression of genes related to chlorophyll degradation, whereas *OsCGA1* knockdown resulted in reduced chlorophyll content [20,21]. In poplar, overexpression of *GNC* dramatically accelerated plant growth, chlorophyll accumulation, and photosynthesis, whereas its CRISPR-knockout lines showed retarded development [22]. Therefore, the significant involvement of the GATA family proteins in chlorophyll synthesis and various other biological processes necessitates a more in-depth examination of their roles and functions involvement in *Eucalyptus*.

In this study, we provide a complete analysis of the numbers, phylogeny, conserved motifs, exon–intron structure, *cis*-elements of the promoters, chromosome distribution, and expression of *GATA* genes in the published genome of *Eucalyptus grandis* (JGI, 2014) to better understand the dynamics of *GATA* gene evolution in *Eucalyptus* and facilitate future study on this critical TF family.

## 2. Results

### 2.1. Identification and Phylogenetic Analysis of Eucalyptus GATA Transcription Factors

A total of 23 genes were identified on the basis of the functional annotation in the *Eucalyptus grandis* genome. The length of GATA proteins ranged from 66 to 545 amino acids (Table S1). The isoelectric point of the proteins ranged from 4.87 to 10.61, and the molecular weight ranged from 72.96 kDa to 604.67 kDa.

**Figure 1:**
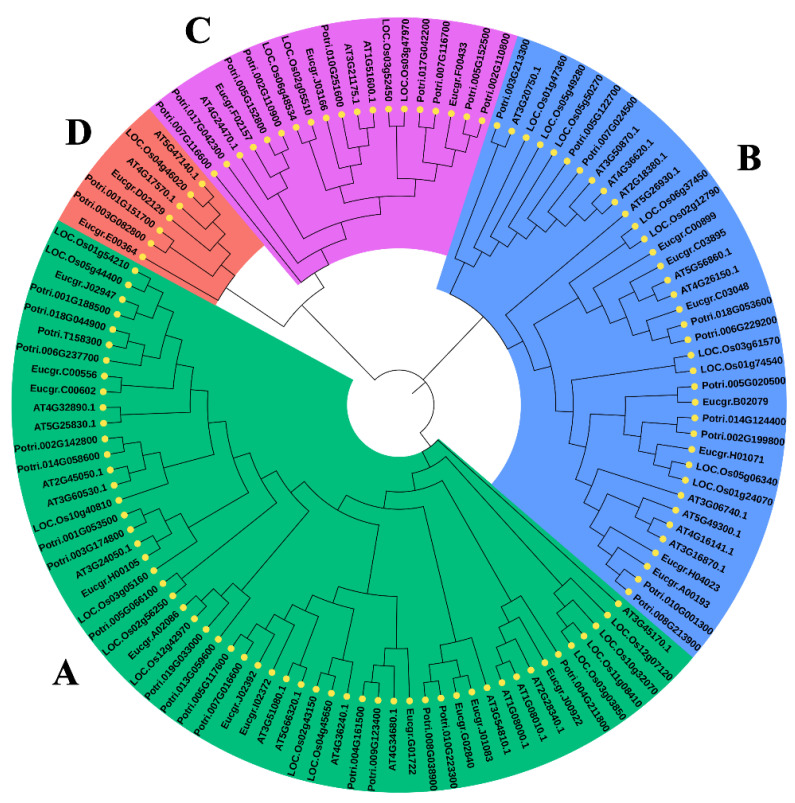
Phylogenetic relationships between GATA proteins from *Arabidopsis*, poplar, rice, and *Eucalyptus*. The GATA members of *Eucalyptus grandis* were classified into four subfamilies together with the homologous genes of other plants. A, B, C, and D represent the four subfamilies, respectively.

The protein sequences encoded by *GATA* genes were used to construct the phylogenetic tree to determine the evolutionary relationship between them. Results showed clustering of GATA proteins of *Arabidopsis*, *Populus*, rice, and *Eucalyptus* into four subfamilies (Figure 1). Preliminary classification of the phylogeny according to the four subfamilies identified by analysis of the GATA protein family from four species displayed good agreement with the topology in our study. Subfamily A was the largest, comprising 54 proteins, followed by 37, 18, and 7 GATA proteins belonging to Subfamilies B, C, and D, respectively. All *Eucalyptus* GATA members formed groups with GATA members of other species in each subfamily. In subfamilies, the gene phylogeny roughly followed species phylogeny, with *Populus* genes being closely related to the *Eucalyptus* genes. These genes may have similar evolutionary background and functions in woody trees.

### 2.2. Gene Structure and Conserved Motifs of GATA Genes

**Figure 2:**
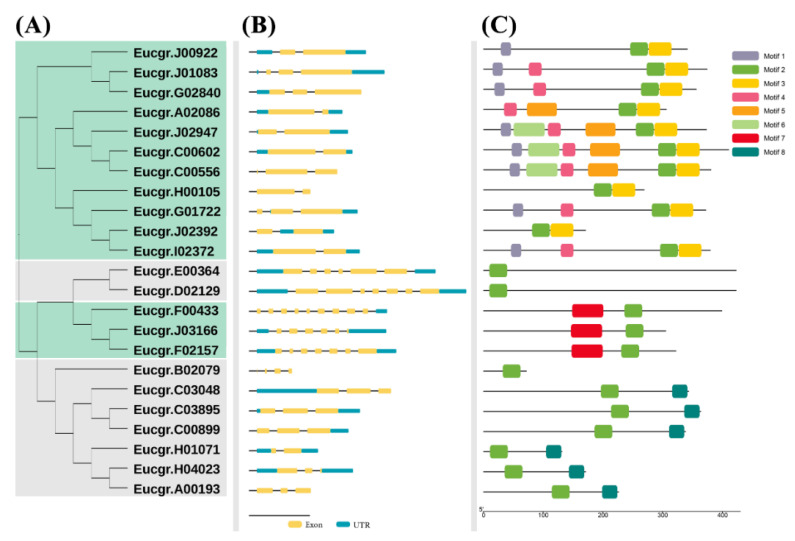
Predicted *Eucalyptus grandis* GATA (*EgrGATA*) protein phylogeny, conserved amino acid motifs, and gene structures. (**A**) Unrooted tree of *EgrGATA* proteins based on maximum-likelihood method. (**B**) Position of exons and UTRs in the *EgrGATA* gene models (yellow line represents exons, and blue line represents UTRs). (**C**) Composition and distribution of overrepresented amino acid motifs (sequences of conserved motifs are given in Figure S1).

The relationship between 23 *Eucalyptus GATA* genes was determined by the construction of a phylogenetic tree using the CDS sequences. To elucidate the structural characteristics of the *EgrGATA* genes, the exon–intron structures and conserved motifs were analyzed (Figure 2). Genes of Subfamilies A and B contained two to three exons, whereas those of Subfamilies C and D contained five to seven exons.

Three genes of Subfamily A, *Eucgr.J01083*, *Eucgr.G02840*, and *Eucgr.G01722,* had three exons, whereas the other eight genes had only two exons. Only *Eucgr.H01701* had two exons in Subfamily B, while the other six genes had three exons. The length and distribution of exons in the same subfamily were also relatively similar. Compared with the other three subfamilies, the length of exons in Subfamily C is shorter (1–1.5 kb). Significant variations in the number and distribution of exons suggest that distinct subfamilies have different evolutionary origins and chromosomal structural variations.

In the EgrGATA proteins, eight motifs were identified by the Multiple EM for Motif Elicitation (MEME) programs and defined as Motifs 1 to 8 (Figure 2C). The detailed sequence of each motif is provided in Figure S1. Motif 2, like the GATA domain, was detected in all EgrGATA proteins. In detail, Motifs 1, 3, and 4 were mainly involved in Subfamily A. Except for Motif 2, no other motifs were recognized in Subfamily B. Motifs 7 and 8 were identified in Subfamilies C and D, respectively. In Subfamily C, Motif 5 was located upstream of Motif 1. Proteins belonging to different subfamilies were found to have similar motif locations. In Subfamily A, Motifs 2 and 3 were located at the C-terminal, and Motif 2 was present upstream of Motif 3. Motif 1 was located at the N-terminal in proteins of Subfamily A, except for those encoded by *Eucgr.A02086* and *Eucgr.H00105*. Proteins from different subfamilies had different motifs, but proteins from the same subfamily had similar motifs.

**Figure 3:**
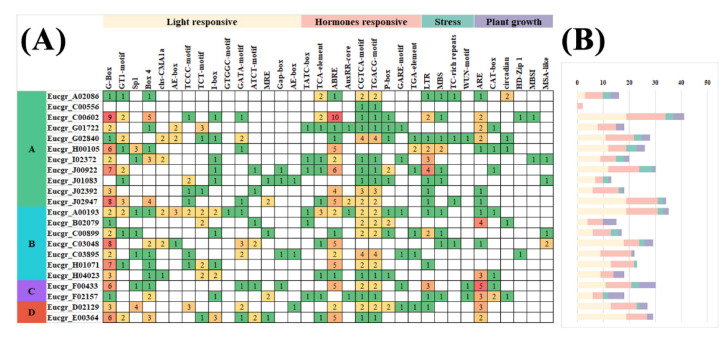
Analysis of *cis*-elements in the promoters of *EgrGATA* genes. (**A**) Overview of the types and numbers of *cis*-elements of the four subfamilies identified from the PLANTCARE database. (**B**) The number of light-responsive, hormone-responsive, stress-, and plant-growth-related elements present in the promoter region of each gene.

To understand gene regulation and function, it is necessary to identify putative *cis*-elements in promoter sequences of *EgrGATA* genes. The identified *cis*-elements were classified into four categories, including light-responsive, hormone-responsive, stress-responsive, and plant growth (Figure 3). There are 15 *cis*-elements that belong to the light-responsive category, such as G-box, GT1-motif, Sp1, Box 4, AE-box, GATA-motif, MRE, and AE-box. Among them, G-box is present in promoter regions of almost all *EgrGATA* genes, except for *Eucgr.C00556* and *Eucgr.J01083*. The hormone-responsive category contained TATC-box (gibberellin-responsive), TCA-element (salicylic-acid-responsive), ABRE (abscisic-acid-responsive), AuxRR-core (auxin-responsive), CGTCA-motif (Methyl jasmonate-responsive), TGACG-motif (Methyl jasmonate-responsive), P-box (gibberellin-responsive), GARE-motif (gibberellin-responsive), and TGA-element (auxin-responsive).

### 2.3. Chromosomal Localization and Collinearity Analysis

**Figure 4:**
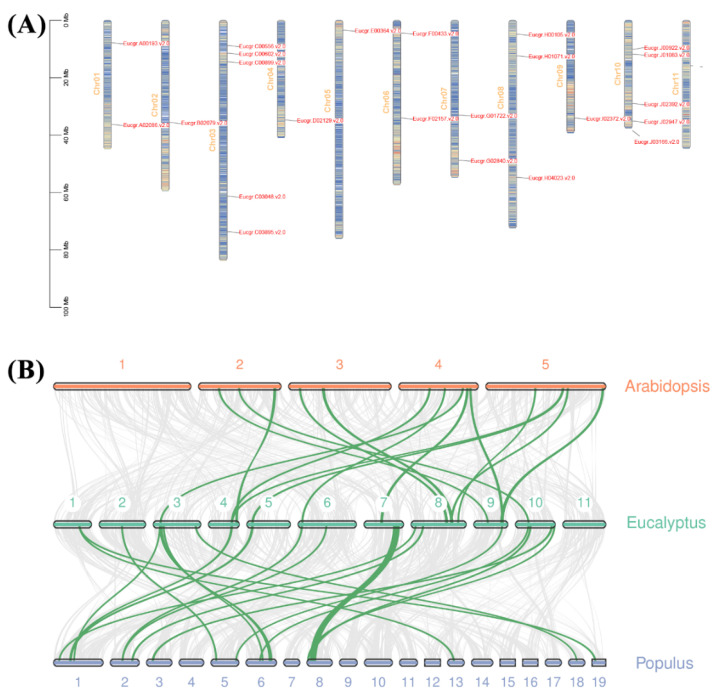
Distribution of *EgrGATA* genes on the chromosomes of *Eucalyptus grandis* and synteny analysis of GATA genes. (**A**) Chromosomal distributions of *EgrGATA* genes. Color gradient from blue to red on the chromosomes indicates low to high gene density, respectively. (**B**) Synteny analyses of *GATA* genes between *Eucalyptus grandis* and other module plant species (*A. thaliana* and *P. trichocarpa*). Green lines indicate the collinear blocks within *Eucalyptus* and other plant genomes.

According to the *EgrGATA* annotation information, 23 genes were mapped to 10 chromosomes of *Eucalyptus*, and GATA family genes were not identified on Chr11 (Figure 4A). The distribution of the *Eucalyptus GATA* gene family on chromosomes is uneven. Gene number or density of the chromosome has no biological relevance to *EgrGATA* distribution. Chromosomes 5 and 10 contained the largest number of *EgrGATA* genes, and Chromosome 8 had three *EgrGATA* genes. The length of Chromosome 5 is second only to that of Chromosome 3, but it contains only one *GATA* gene.

To better understand the evolutionary relationships of GATA from *Arabidopsis*, *Populus*, and *Eucalyptus grandis*, we constructed a collinearity chart (Figure 4B). There are 14 and 25 *GATA* genes in *Arabidopsis* and poplar, respectively, which have a collinearity relationship with *EgrGATA*s. Among them, 13 genes are shared by *Arabidopsis* and poplar, which indicates that the GATA family has lost and reconstructed sequences in woody plants during evolution. Furthermore, the Ka/Ks ratio was calculated between *Eucalyptus grandis* and other species (Table S2). These results suggest that most homologous genes are under purifying selection in *Eucalyptus*.

### 2.4. Tissue-Specific Expression Patterns of GATA Genes

**Figure 5:**
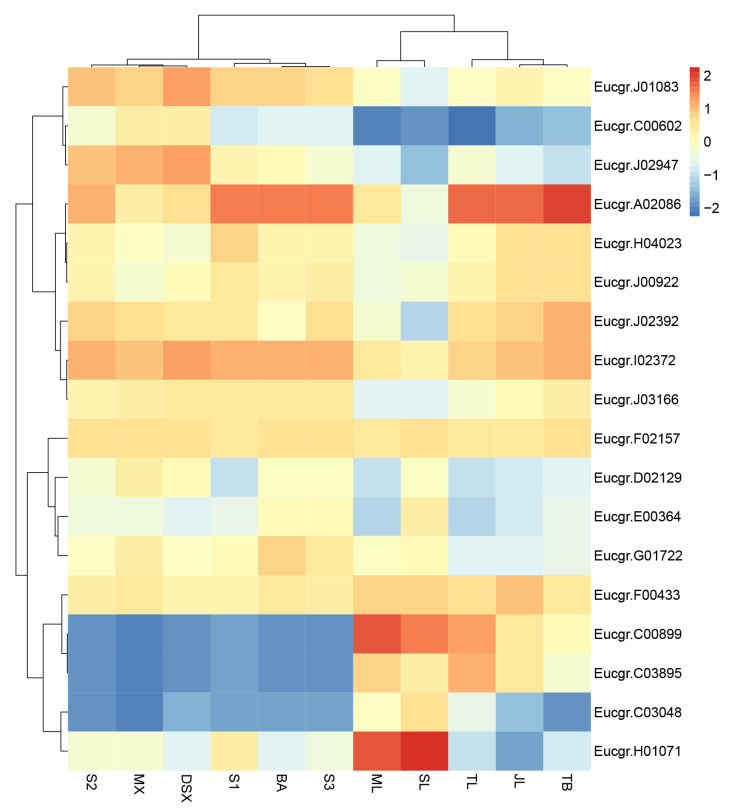
Tissue-specific gene expression patterns of 22 *EgrGATA* genes. The expression patterns of genes in terminal bud (TB), juvenile leaves (JL), transition leaves (TL), mature leaves (ML), senescent leaves (SL), young stem (S1), transition stem (S2), mature stem (S3), bark (BA), developing secondary xylem (DSX), and mature xylem (MX). The red and blue colors indicate the high and low transcript abundance, respectively. Expressions normalized by log2.

The expression patterns of *EgrGATA* genes were further analyzed based on RNA-seq data of 11 different tissues (terminal bud, spire leaves, transition leaves, mature leaves, senescent leaves, young stem, transition stem, mature stem, bark, developing secondary xylem, and mature xylem). A total of 22 *EurGATA* showed expression in different tissues and exhibited tissue-specific expression (Figure 5). Heat map and hierarchical cluster analysis showed that expression patterns of these genes were different in different tissues. In this study, *Eucgr.C00602* and *Eucgr.J02947* were highly expressed in the stem, while the other four *GATA* genes (*Eucgr.C00899*, *Eucgr.C03895*, *Eucgr.C03048*, and *Eucgr.H01071*) had high expression levels in the leaves. However, the expression levels of some *EurGATA* genes were similar in several tissues, such as *Eucgr.F02157*, *Eucgr.F00433*, *Eucgr.J00922*, and *Eucgr.I02372*.

### 2.5. GATA Genes Are Involved in Chlorophyll Synthesis

**Figure 6:**
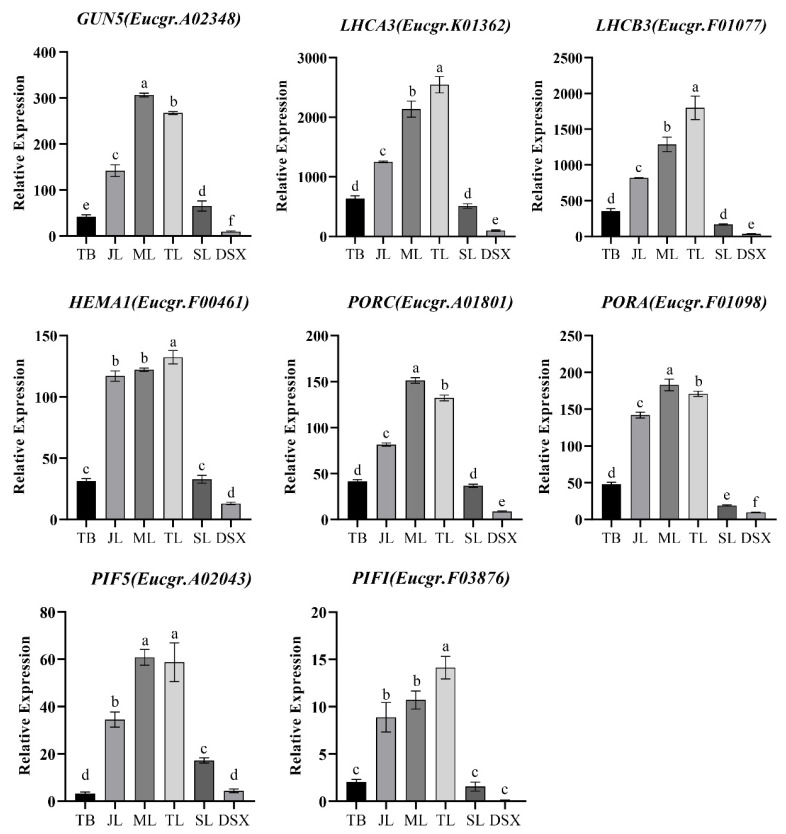
The qRT-PCR analyses of EgrGATA genes in leaves and stems. The qRT-PCR analyses of eight genes expressed in terminal bud (TB), spire leaves (SL), transition leaves (TL), mature leaves (ML), senescent leaves (SL), and developing secondary xylem (DSX). *Photosystem I light-harvesting complex gene 3* (*LHCA3*). The different lowercase letters show statistically significant differences by the Tukey-Kramer multiple comparison test at *p* < 0.05.

**Figure 7:**
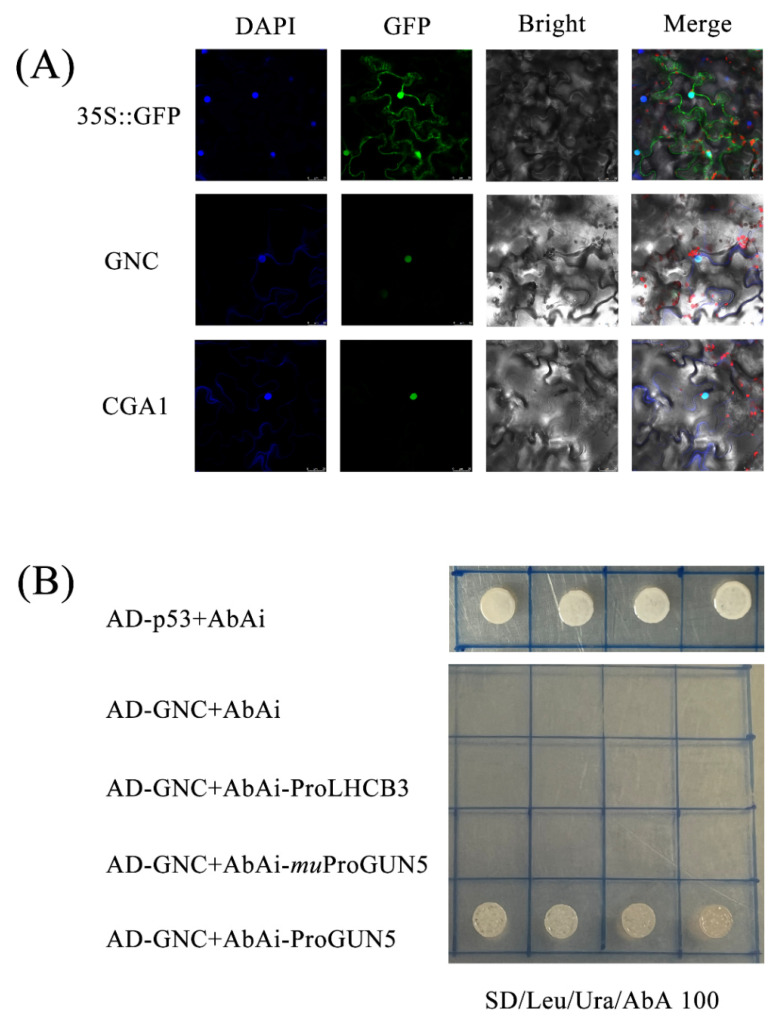
*EurGNC* is a nuclear-localized transcriptional regulator. (**A**) Subcellular localization of *EurGNC* and *EurCGA1* proteins. Fluorescence signals of GFP were detected in tobacco leaf epidermal cells. Left panel, DAPI and GFP image; middle panel, bright field; and right panel, merging of GFP and bright field. Bar, 25 µm. (**B**) Yeast one-hybrid assay to detect whether *EurGNC* regulates downstream genes (*EurGUN5* and *EurLHCB3*). AbA (100 ng/L) was used to repress the autoactivation. Empty pGADT7 (AD) and AbAi were used as negative controls, while pGADT7-p53 and pAbAi-53 were used as positive controls.

To investigate the relationship between *EgrGATA*s and the genes involved in chlorophyll synthesis, the transcription levels of those genes were detected by qRT-PCR (Figure 6). Like *EurGNC* (*GATA, NITRATE-INDUCIBLE*, *CARBON-METABOLISM-INVOLVED*), *EurGUN5* (*GENOMES UNCOUPLED 5*) was also highly expressed in leaves and had low expression in stems. To detect the subcellular localization of the *EurGNC* and *EurCGA1* (*GNC-LIKE/CYTOKININ-RESPON-SIVE GATA FACTOR1*), gene sequences were fused with the GFP reporter gene downstream of the CaMV 35S promoter. In *Nicotiana benthamiana*, reporter gene expression showed that *GNC* and *CGA1*-GFP fusion protein was predominant in the nucleus, indicating that *CGA1* and *GNC* are nuclear-localized transcription factors (Figure 7A). The close relationship and similar expression pattern indicated that *EurGNC* may activate *GUN5* or *light-harvesting chlorophyll B-binding protein 3* (*LHCB3*); thus, it controls the expression of a series of chlorophyll-related genes.

To verify the interaction between *EurGNC* and the upregulated *GUN5*, a yeast Y1H assay was conducted. *EurGNC* could not directly bind to the promoter of *LHCB3* in the Y1H assay (Figure 7B), but it can bind to *EurGUN5*. *EurGNC* bound to the promoter fragments containing the *cis*-element (GATC), whereas it could not bind to the promoter fragments containing the mutated *cis*-element GATA.

## 3. Discussion

In previous studies, GATA family genes have been demonstrated to affect growth regulation, defense response, and stress tolerance in plants [10,23,24,25]. To date, *GATA* family genes have been identified and characterized in *A. thaliana*, rice [2], and *Populus* [7]. However, as a representative plant of the order *Myrtales*, genome-wide analysis of the GATA family has not been performed in *Eucalyptus grandis*, and the regulatory function of *EgrGATA* genes remains unclear. In this study, we systematically analyzed 23 *GATA* genes in the released *Eucalyptus grandis* genome. With the continuous development of gene-editing technology in *Eucalyptus* [26], research on growth-related TFs will provide candidate genes for genome editing and precision plant breeding.

### 3.1. Phylogenetic Analysis of EgrGATAs

Based on phylogenetic analysis, *EgrGATA* genes were classified into four subfamilies (Figure 1), which is consistent with classification in other plant species, such as that of *A. thaliana* and poplar. Therefore, *GATA* might have been present in the common ancestor of eudicotyledons. Surprisingly, *OsGATA* (*LOC.Os04g46020*) appeared on the branch of Subfamily D, which is contrary to the previous research which suggests, that Subfamily D *GATA*s originated after the differentiation of monocotyledon and dicotyledon, and no *OsGATA*s belonged to Subfamily D [2,22]. However, phylogenetic analyses of pepper, *Gossypium*, soybean, and cucumber revealed that *OsGATA*s occur in the Subfamily D branch [6,27,28,29]. Therefore, we speculated that due to the increase in the accuracy of genome sequencing, evolutionary analysis of the GATA family revealed that Subfamilies A, B, C, and D originated before the differentiation of monocots and dicots.

The number of *GATA*s identified in the *Arabidopsis* and poplar genomes were 30 and 39, respectively. Only 23 genes were identified in *Eucalyptus*, which belonged to the four subfamilies. We suspected that *EgrGATA*s were lost during evolution but found that sufficient genetic diversity was retained; a similar evolutionary loss event occurred in the *WRKY* transcription factor family [30]. According to the analysis of exon structure and motifs of *EgrGATA*s, different *EgrGATA*s from the same subfamily have similar numbers and patterns and tend to share the same evolutionary origins.

In the process of evolution, segmental and tandem duplications contributed to the formation and rapid expansion of gene families [31,32]. Previous research revealed that 28 *EgrWRKY*s participated in tandem duplication events without segmental duplication events in *E. grandis* [33]. Tandem duplication has occurred in several gene families, such as AP2, GRAS, MYB, *p*-coumarate 3-hydroxylase (*C3H*), caffeate/5-hydroxyferulate O-methyltransferase (*COMT*), and *ROS* [34,35,36,37]. Although *EgrGATA*s are slightly contracted compared to GATAs of most angiosperms studied hitherto, they lack traces of duplication events (Figure 2 and Figure 4), and a similar conclusion was reached upon the analysis of the *Eucalyptus ARF* family [38].

### 3.2. Structural and Functional Variation of EgrGATAs

The exon structures and motifs of *EgrGATA*s in the same branch had similar characteristics, thus implying that the genes in the same subfamily may have similar expression patterns and biological functions. During tissue development, the expression levels of *EgrGATA*s (*EgrGATA12* (*Eucgr.C00602*) and *EgrGATA9* (*Eucgr.J02947*)) increase in the xylem of the transition stem, developing secondary xylem, and mature xylem (Figure 5). The *GATA12*(*Potri.006G237700*) was predominantly expressed in developing xylem tissues and involved in the regulation of secondary cell wall component biosynthesis pathways in *Populus trichocarpa* [39]; it indicates a crucial role of *EgrGATA12* in regulation of xylem differentiation. Additionally, the expression of *EgrGNC* (*Eucgr.C03048*) and *CGA1/GNL* (*Eucgr.C00899*) were upregulated in the leaves (Figure 5). In *Arabidopsis*, stomata formation is promoted by overexpression of *GNC* and *CGA1* [40], which also regulate germination, greening, growth, and flowering time [24].

The *cis*-element is the short sequence located in the promoter regions of the genes that could be activated by trans-acting elements to regulate the activity of target genes [41]. The analysis of the *cis*-elements in the *EgrGATA* promoters showed that 21 *EgrGATA*s were related to light response (Figure 3A), and the G-box element was present most frequently in the promoters of those genes. In *Catharanthus roseus*, *CrPIF1* repressed *CrGATA1* promoter activity by binding to G-box elements, and the expression of *CrGATA1* was significantly induced by light [42]. We found numerous *cis*-elements that are hormone-responsive, implying that *EgrGATA*s might form a hub for multiple hormones. Three gibberellic acid (GA)-responsive elements (TATC-box, P-box, and GARE-motif) were found in the promoter of 14 *EgrGATA*s. The promoters of 20 *EgrGATA*s had CGTCA and TGACG elements, which are MeJA-responsive, and the CGTCA-motif induces transcription of CICMO and CIBADH to accelerate the biosynthesis of glycine betaine in watermelon [43]. There are 63 abscisic-acid-responsive elements (ABREs) that span the promoter regions of 18 *EgrGATA*s; these elements, as positive regulators of cold tolerance, could bind with PsnICE1 in poplar [44]. Auxin-responsive element (TGA-element) was a key component for indole-3-acetic acid responsiveness [45], which is bound by ARFs to regulate plant architecture [46]. In our study, TGA-element was found in the promoter of six *EgrGATA*s.

### 3.3. EurGATAs Were Involved in the Biosynthesis of Chlorophyll

The essence of photosynthesis is the absorption and harvesting of light energy by chlorophyll in the plant leaves, and light absorption strongly depends on the chlorophyll content [47]. Studies in several species have documented that the GATA TF family affects chlorophyll synthesis directly or indirectly [19,48,49]. Overexpression of GATA in *Arabidopsi*s increases the chlorophyll content and improves photosynthetic efficiency [49]. In this study, the expression levels of *EurLHCB3* and *EurGUN5* were positively correlated with the expression of *EurGNC* and *EurCGA1* (Figure 4). *GNC* and *CGA1* are the two master transcriptional regulators that could affect chlorophyll synthesis genes (*GUN4*, *HEMA1*, *PORB*, and *PORC*) localized both in the nucleus and chloroplast (GLUTAMATE SYNTHASE (*GLU1/Fd-GOGAT*)) [18]. Y1H assays showed that *GNC* could directly bind to the promoter of *EurGUN5* (Figure 7B), thus revealing that *EurGNC* would directly activate *EurGUN5* to accelerate chlorophyll biosynthesis. In addition, we found that *GNC* could not bind to the mutated *GUN5* promoter sequence (‘GATC’ mutated to ‘GATA’). The GATA gene families were named because they contained ‘GATA’ sequences in the binding elements; however, subsequent studies have found that the GATA family motif can also recognize the GATC sequence [50].

## 4. Materials and Methods

### 4.1. Identification of GATA Family Genes in Eucalyptus

*Arabidopsis thaliana*, *Eucalyptus grandis*, rice, and poplar GATA nucleic acid and protein sequences were retrieved from the PlantTFDB 5.0 (http://planttfdb.cbi.pku.edu.cn, accessed on 5 October 2021). Multispecies GATA data were searched using the Hidden Markov Model (HMM) of HMMER 3.1 to identify whether or not it has the GATA zinc finger domain (PF00320) [51]. The theoretical molecular weight (MW) and isoelectric point (pI) of the encoded proteins were analyzed using the ProtParam tool (https://web.expasy.org/protparam/, accessed on 5 October 2021).

### 4.2. Analysis of Phylogenetic Relationships, Gene Structures, Conserved Motifs, and Protein

Multiple sequence alignment of amino acid sequences was performed using the Multiple Alignment using Fast Fourier Transform tool with default parameters. Subsequently, the results of multiple sequence alignment were trimmed using trimal, and a phylogenetic tree was generated using IQ-TREE. The phylogenetic tree generated was visualized using iTOL (http://itol.embl.de, accessed on 10 October 2021). Using the same method, a phylogenetic tree of *Eucalyptus* GATA proteins was constructed. Gene structure display server (http://gsds.cbi.pku.edu.cn, accessed on 10 October 2021) was used to map coding sequences to genomic sequences and visualize exon–intron structures. *Eucalyptus* GATA sequences were submitted to WebLogo (http://weblogo.berkeley.edu, accessed on 10 October 2021) for online analysis to map conserved structural domains. The genomic DNA sequences (2000 bp) present upstream to the 5′UTR were obtained from the genome of *Eucalyptus grandis* using TBtools. The promoters of *EgrGATA* were submitted to PlantCARE (http://bioinformatics.psb.ugent.be/webtools/plantcare/html, accessed on 15 October 2021) for analysis of *cis*-acting elements. The diagram of *cis*-elements present in the promoter regions was visualized by TBtools.

### 4.3. Chromosomal Location and Cross-Species Collinearity Analysis

The chromosomal locations of the *EgrGATA* genes were derived from the gene annotation file and presented using TBtools. Cross-species collinearity analyses and plotting were performed using MCscan (Python version) of jcvi (v.0.8.12) and annotation files retrieved from Phytozome. TBtools was used to estimate nonsynonymous substitution rate (Ka), synonymous substitution rate (Ks), and Ka/Ks of the paralogous gene pair. When the Ka/Ks ratio was >1, <1, or =1, selection pressure on *EgrGATA* was considered positive, negative, or neutral, respectively.

### 4.4. Expression Profile Analysis of GATA Gene Family

Plants from the Guangxi Dongmen Forest Farm’s *Eucalyptus urophylla* were used in this study. Tissues (terminal bud, spire leaves, transition leaves, mature leaves, senescent leaves, young stem, transition stem, mature stem, bark, developing secondary xylem, mature xylem) from five months seedlings were used for analysis of tissue-specific expression (Figure S2). The total RNA was extracted and purified using TRIzol reagent (Invitrogen, Carlsbad, CA, USA), following the manufacturer’s instructions. Paired-end sequencing was performed on an Illumina Novaseq™ 6000, following the vendor’s recommended protocol. The FASTQ data were filtered using fastx-toolkit (http://hannonlab.cshl.edu/fastx_toolkit/index.html, accessed on 15 October 2021) based on a quality score criterion of Q20 and were then inspected with the FastQC program (http://www.bioinformatics.babraham.ac.uk/projects/fastqc/, accessed on 15 October 2021). Fragments per kilobase of exon per million fragments mapped (FPKM) values of isoform genes were calculated by Cufflinks.

### 4.5. RNA Isolation and Quantitative Real-Time PCR (qRT-PCR) Validation

Total RNA from *E. urophylla* of different stages was the same as 4.4 used. The first-strand cDNA was generated using the FastQuant RT kit (Tiangen Biotech Co., Ltd., Beijing, China) with gDNase. A SuperReal PreMix Plus (SYBR Green) kit (Tiangen Biotech, Beijing, China) was used to prepare qRT-PCR reaction mixtures. Reactions were performed on the Applied Biosystems 7500 Fast Systems (AB Ltd., Lincoln, NE, USA) with *Eucgr.H04673* as the reference gene. Primer sequences are listed in Table S3.

### 4.6. Cloning of EurGNC and EurCGA1 Coding Sequence (CDS) and Promoters of EurGUN5 and EurLHCB3

The full-length CDS of *EurGNC* and *EurCGA1* were amplified by PCR using cDNA from 2.5. Promoters of *EurGUN5* and *EurLHCB3* were amplified from the genomic DNA.

### 4.7. Subcellular Location Analyses

To validate the position of GATA, the full-length CDS without a stop codon was cloned into the pBI121-eGFP vector. The verified plasmid vector was transformed into an LB4404 Agrobacterium competent cell, which was then used to invade and transform *Nicotiana benthamiana*. After 2 days, the fluorescence was detected using Olympus FV1000 confocal laser-scanning microscope (Olympus, Tokyo, Japan). GFP fluorescence was detected using an excitation filter at 450–490 nm.

### 4.8. Yeast One-Hybrid Assay

For yeast one-hybrid assay (Y1H), promoter fragments of *EurGUN5* and *EurLHCB3* were cloned into the pAbAi vector (Clontech, Beijing, China), and inserted upstream of the AbAr reporter gene (AUR1-C). The primers used are listed in Table S1. The ORFs of *EurGNC* were inserted into the pGADT7 vector to generate recombinant pGAD-*EurGNC* constructs. The pGADT7 and pAbAi-*LHCB3* were used to represent negative controls. Pairs of plasmids were integrated into the yeast strain Y1HGold as described [9] and cultured on synthetic defined (SD) medium without Leu/Ura. After 3 days, the transformants were confirmed by colony PCR analysis. The resulting co-transformed yeast strains were then plated on the SD/-Leu/-Ura medium containing 50 ng/mL aureobasidin A to confirm TF–promoter interactions.

## 5. Conclusions

In conclusion, the *GATA* TFs are of critical importance for *Eucalyptus* development, and hence bear critical potential for the improvement of this highly economically relevant woody plant. Based on the phylogenetic and gene structure analyses, these genes were categorized into four subfamilies, which was consistent with the previously reported GATA families. Our study provides new insights into the evolution of the *GATA* gene family in angiosperms. *Cis*-element prediction and expression trends among the subfamilies of *EgrGATA*s showed they are modulated by various hormones and are involved in a range of physiological processes in different tissues. Moreover, qRT-PCR and Y1H assays demonstrated that *EgrGNC* acts as an activator and enhances the expression of *EgrGUN5*, which facilitates chlorophyll accumulation. Finally, our systematic analysis provides essential and beneficial knowledge for researchers to further study the function of different GATA TF family members in *Eucalyptus* and other plants.

## Data Availability

The data presented in this study are available in Supplementary Materials.

## Supplementary Materials

The following supporting information can be downloaded at: www.mdpi.com/article/10.3390/ijms23095251/s1.
