# Supplementary figures and images for: Genome-Wide Identification of the Eucalyptus urophylla GATA Gene Family and Its Diverse Roles in Chlorophyll Biosynthesis
Source: Int J Mol Sci. 2022 May 8;23(9):5251. doi: 10.3390/ijms23095251 (PMC9102942; doi:10.3390/ijms23095251)

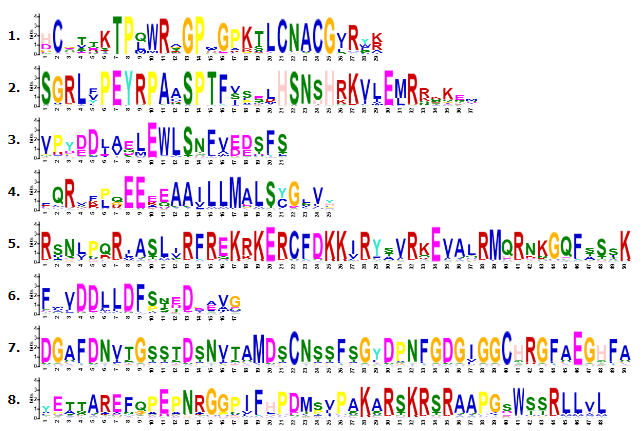

Supplement: Supplementary file 1 [file ijms-23-05251-s001.zip › Figure S1. Sequences logos of domains of GATA proteins.png]

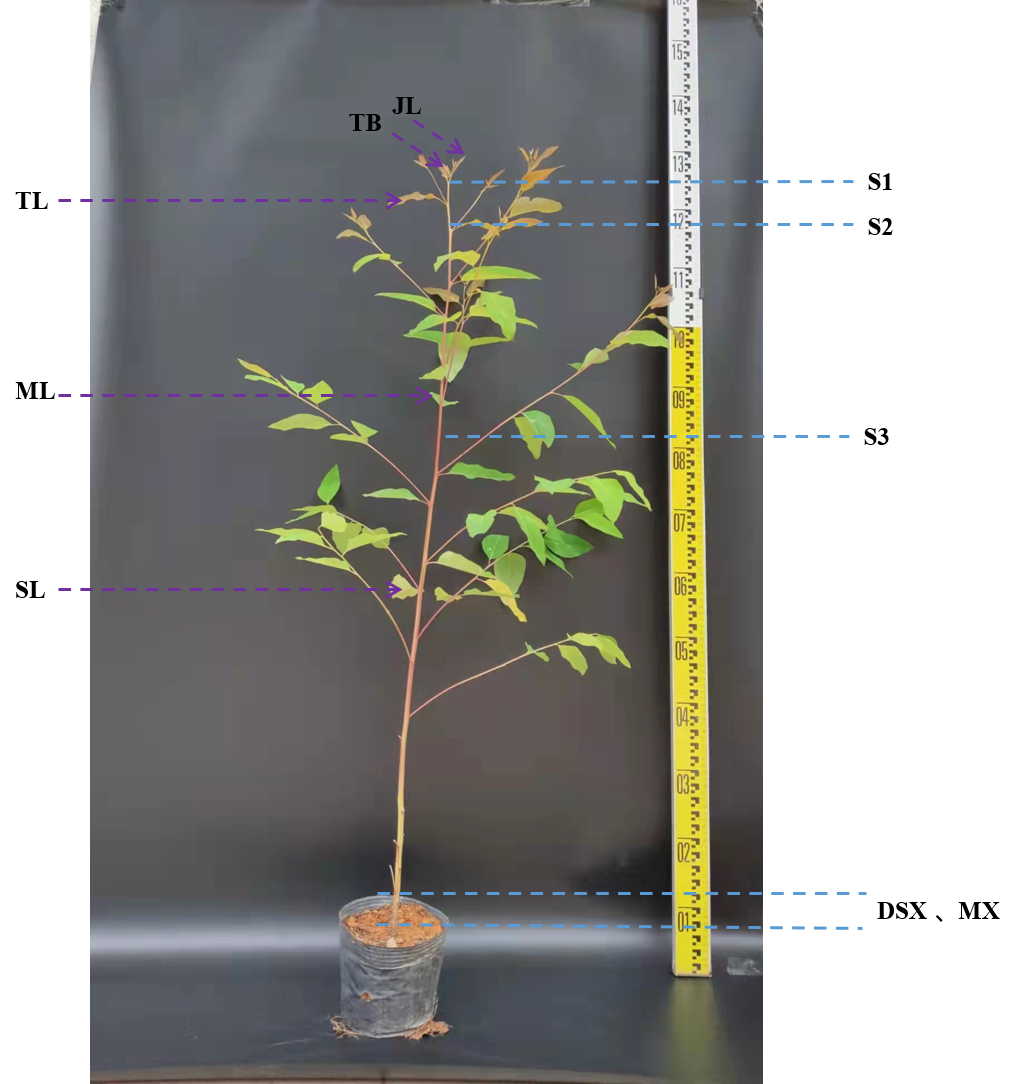

Supplement: Supplementary file 1 [file ijms-23-05251-s001.zip › Figure S2. Sample schematics.tif]
